# A Low-Cost Microfluidic and Optically Transparent Water Antenna with Frequency-Tuning Characteristics

**DOI:** 10.3390/mi14112052

**Published:** 2023-11-01

**Authors:** Abdullah Abdullah, Syed Imran Hussain Shah, Sakobyly Kiv, Jinwoo Ho, Sungjoon Lim

**Affiliations:** Department of Electrical and Electronics Engineering, Chung Ang University, Seoul 06974, Republic of Korea; shahabdullahuet@gmail.com (A.A.); kiv.sakobyly168@gmail.com (S.K.); ghwlsdn221@cau.ac.kr (J.H.)

**Keywords:** transparent antennas, water-based antenna, wideband antenna, frequency reconfigurable antenna

## Abstract

In this study, a novel microfluidic frequency reconfigurable and optically transparent water antenna is designed using three-dimensional (3D) printing technology. The proposed antenna consists of three distinct parts, including a circularly shaped distilled water ground, a sea water-based circular segmented radiator, and a circularly shaped distilled water-based load, all ingeniously constructed from transparent resin material. The presented antenna is excited by a disk-loaded probe. The frequency of the antenna can be easily tuned by filling and emptying/evacuating sea water from the multisegmented radiator. The radiator consists of three segments with different radii, and each segment has a different resonant frequency. When the radiator is filled, the antenna resonates at the frequency of the segment that is filled. When all the radiator segments are filled, the antenna operates at the resonant frequency of 2.4 GHz and possesses an impedance bandwidth of 1.05 GHz (40%) in the range of 2.10–3.15 GHz. By filling different radiator segments, the frequency could be tuned from 2.4 to 2.6 GHz. In addition to the frequency-switching characteristics, the proposed antenna exhibits high simulated radiation efficiency (with a peak performance reaching 95%) and attains a maximum realized gain of 3.8 dBi at 2.9 GHz. The proposed antenna integrates water as its predominant constituent, which is easily available, thereby achieving cost-effectiveness, compactness, and transparency characteristics; it also has the potential to be utilized in future applications, involving transparent and flexible electronics.

## 1. Introduction

Most wireless communication systems use antennas made of conductive metals, like copper and rigid dielectrics. However, the development of new materials, such as papers, conductive oxides, conductive inks, carbon nanotubes, liquid metal alloy, and liquid dielectrics, has led to the creation of a new type of antenna that is flexible, transparent, and reconfigurable [1]. Many transparent materials, such as Indium-tin-oxide (ITO), fluorine-doped tin oxide (FTO), and Indium-zinc-tin oxide (IZTO), are associated with complex fabrication processes and increased prices, rendering them inaccessible in terms of availability [2,3]. Frequency tuning and flexibility are excellent features of liquid materials used in antenna development. These materials can be easily enclosed in three-dimensional (3D) printed containers from plastic, resin, or glass; by changing the amount of liquid, they can produce frequency reconfigurability. Water is a promising material for the design and fabrication of transparent antennas, but it is challenging to create efficient and compact water-based antennas. Water is an ideal material for transparent and reconfigurable antennas because it is flexible, easily accessible, highly transparent, environmentally friendly, and nontoxic. Water-based antennas can be divided into three types: distilled or pure water antennas, sea water antennas, and hybrid antennas. Distilled or pure water antennas, sea water antennas, and combinations of both sea and distilled water antennas are referred to as hybrid antennas. Pure water possesses a high dielectric constant and low-loss tangent at low frequencies and has a very-low-electrical conductivity, while seawater has good electrical conductivity which depends on the amount of salt contents present in the water and changes from place to place. A salt-saturated water solution can achieve electrical conductivity values up to 20 S/m [4]. In the literature, various transparent antennas have been developed using water. Most of them are water-based monopole antennas but a few patch antennas have also been developed. In [5], a coaxial, dual-tube hybrid monopole antenna was proposed with a metal ground. This antenna achieved a higher radiation response by inserting distilled water as a dielectric load in the sea-water tube. This enhanced radiation efficiency can be attributed to two key factors. Firstly, the height of the Teflon base, which separates the water and the metal ground, effectively contains a significant portion of electromagnetic energy, thereby reducing losses associated with water. Secondly, the distilled water within the monopole primarily serves as a load, thus minimizing dielectric losses, as opposed to being the primary source of radiation. The combination of various modes within the hybrid coaxial structure results in an impedance bandwidth with |S_11_| < −15 dB that spans 57.27%. Moreover, the radiation efficiency falls within the range of 52% to 84% across the operational bandwidth. However, this antenna is not fully transparent because of the metal ground at its bottom part. A first water-patch antenna with a metallic ground was proposed in [6]. This antenna exhibited a narrow bandwidth performance, and the metallic ground restricted the transparency of the entire antenna. In [7], a compact transparent water antenna was proposed with the highest relative operating bandwidth (82.5%) of any transparent antenna reported to date. The overall size of this antenna was reduced by combining a fan-shaped radiator with a top disk distilled water load. However, the antenna had no capability of frequency tuning functioning although the antenna was able to achieve good efficiency and bandwidth by utilizing water as its major part. A circularly polarized water patch antenna with a distilled water patch and a ground was proposed in [8]. Two transparent water patch antennas with L-shaped water [9] and metallic feedings were also designed [10]. Both antennas worked as conventional metallic patch antennas, which constitutes an excellent feature of these antennas. Another transparent dielectric-loaded reconfigurable antenna was proposed in [11]. This antenna used a transparent dielectric loading, a water layer, and a varactor diode. By controlling the direct current (DC) voltage over the varactor diode, the resonant frequency was tuned. However, this antenna also used a metallic ground that resulted in diminished transparency. In reference [12], a four element optically transparent MIMO antenna is designed for mm-wave 5G applications. The antenna has used plexiglass as a substrate and AgHT-8 as conductive parts for the patch and ground layer. The antenna can achieve a good radiation efficiency (>75%) with a dual frequency band characteristic, 24.10–27.18 GHz, and 33–44.13 GHz. Similarly, another two ports UWB MIMO antenna is proposed in [13] for automotive applications. The patch and ground in this antenna are made of ITO/FTO as conducting parts while soda-lime glass is used as a substrate. The antenna shows a wide band operation from 2.4 to 11 GHz, with a peak radiation efficiency of 60%. Both MIMO antennas show excellent MIMO performance of ECC and DG with good isolation between the ports. Another transparent water antenna with omnidirectional conical beam radiation patterns was presented in [14]. This antenna uses coaxial feeding with a disk load and a thick air substrate to obtain a wide impedance bandwidth. The antenna has the notable radiation efficiency of 57–82% is due to the water patch antenna’s operation mechanism is like that of a patch antenna. Electromagnetic waves predominantly remain confined to the central region between the two water patch layers, resulting in minimum radiation loss attributable to water; however, it lacks frequency reconfiguration capability. In [15], a transparent dual band antenna with µ-negative material loading for smart devices is proposed. A distilled water dielectric resonator antenna (DRA) with high efficiency and a broad frequency tuning range was presented in [16]. This antenna had a separate control system of mechanical switches and a microfluidic pump which made its structure bulky and limited its practical applications in certain systems.

Some of the water-based antennas with different functionalities [17,18,19], flexible antennas [20], liquid based antennas [21,22], transparent MIMO antennas [23,24], flexible electronically tunable antennas using polymer-embedded conductive fabric [25]. A pattern reconfigurable microstrip antenna based on electromechanical coupling [26], a high gain square tooth-enabled metamaterial superstrate microstrip patch antenna [27] and a study of microstrip antenna made of transparent ITO films is presented in [28]. All these works are related to implementing different materials for the antenna applications.

In this study, we propose a frequency-reconfigurable and optically transparent water monopole patch antenna. The patch and ground were both made of water enclosed in a transparent 3D-printed design. Distilled water is used for the ground, while sea water is used for the patch due to its higher conductivity. The seawater patch further loaded by a distilled water container to improve the impedance bandwidth of the proposed antenna. The antenna was excited using a disk-loaded probe placed underneath the sea water patch. The patch was split into three sections so that sea water could be filled independently through holes constructed in distilled water top load to reconfigure the operating frequency of the antenna. The proposed transparent antenna is less expensive and easier to make than previous designs because it uses water. The substrate between the two water layers is air, making the antenna almost entirely optically transparent. The only opaque component is the metallic probe feed. In the following parts, we present a discussion on the parametric study of the proposed antenna, that was conducted using the proposed antenna model.

## 2. Antenna Geometry

**Figure 1:**
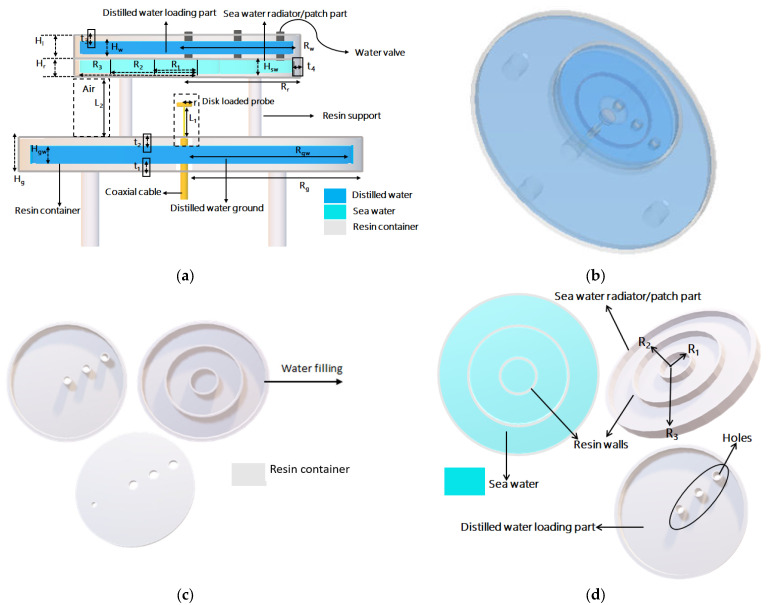
Configuration of the proposed microfluidic water antenna. (**a**) Schematic front view. (**b**) Prospective view. Configuration of the proposed antenna’s segmented radiator and loading part (**c**) Inner front view (**d**) Prospective view.

The structures and perspective of the proposed microfluidic water antenna are shown in Figure 1. The detailed structure dimensions are listed in Table 1. The antenna has three distinct parts, a ground, a radiator or patch, and a loading section. The ground is a circular container with a radius of R_g_, and it is filled with distilled water at a certain height. This distilled water ground provides transparency to the entire antenna structure and acts as a reflector. The radiator has three sections (each having an outer radius R_r_ filled with sea water with a conductivity and loss tangent of 4 S/m and 81, respectively. This radiator is divided into three circular segments with radii R_1_ = 7 mm, R_2_ = 20 mm, and R_3_ = 33 mm separated by fixed resin walls to avoid any flow of water from one segment to another, as illustrated in Figure 1b. Each of these segments has a separate water injection system through the top loading part. The radiator’s injection system consists of three separate narrow water valves, constructed in the loading part. The sea water can be easily injected and evacuated using these water valves. This radiator works like a patch in conventional patch antennas, and it is placed at a height (L_2_ = 15.5 mm) from the ground. The medium between the ground and the radiator is air, which is a low-cost optically transparent material, and a disk-loaded feeding probe is placed between them. The antenna can be structured easily by placing the loading part on top of radiator and then enclosing the loading part, through the head or cap with water holes for different radiator segments as well as for itself filling with distilled water.

All containers are made of transparent resin with a permittivity of 2.5 and loss tangent of 0.04; thus, the entire antenna structure is constructed from transparent material, water, and resin. To make the antenna structure stable, four separate cylindrical resin supports are used for both ground and radiator sections. The proposed antenna uses coaxial feeding and a disk-loaded probe on the top leading to a limited part with a nontransparent material. The inner and outer conductors of the coaxial cable are designed to pass through the distilled water ground and reach into the air medium, thus ensuring the propagation of electromagnetic waves in the medium.

## 3. Results and Discussion

**Figure 2:**
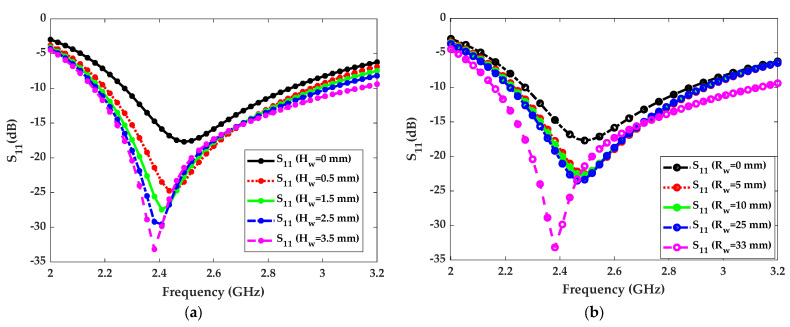
Distilled water loading effects on simulated S_11_ parameters. (**a**) Changes in the height of water. (**b**) Changes in the radius of water.

The simulated and measured results for the proposed water antenna are discussed in this section. For simulations, the software HFSS was used while the S_11_ parameter measurements were conducted using an N9951A Keysight vector network analyzer. As the proposed antenna is a hybrid antenna utilizing distilled water and sea water simultaneously, a detailed parametric study was conducted for both types of water. For better understanding, we first examined the effects of distilled water as the loading part. Distilled water induces no electric currents, and it becomes lossy at frequencies beyond 2 GHz. Distilled water was used in the loading section because of its lower conductivity and higher dielectric response. This helped to achieve a wide impedance bandwidth for the proposed antenna. To optimize the impedance bandwidth of the water-patch antenna, the height of the distilled water was varied from 0 to 3.5 mm. As shown in Figure 2a, the impedance bandwidth increased as a function of the height/thickness of the distilled water. Based on this simulation study, the optimized water level of 3.5 mm was selected for the final design. Similarly, the radius of the distilled water loading varied from 0 to 33 mm. The effects of radius changes on S_11_ are shown in Figure 2b. As shown, the impedance bandwidth increased as a function of the radius of the distilled water loading, and the best result was achieved at 33 mm, which was selected for the final design.

**Figure 3:**
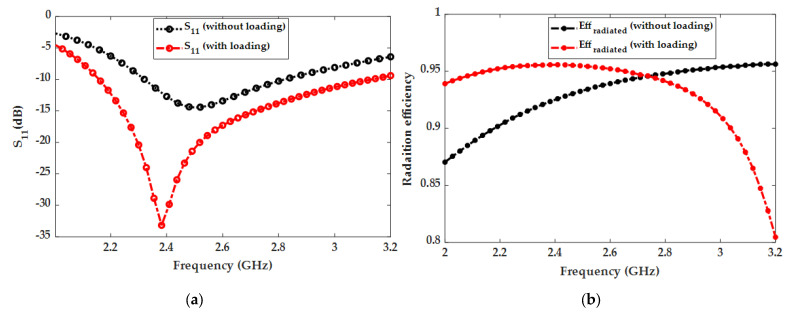
Effects of distilled water loading on (**a**) the S_11_ parameter and (**b**) efficiency.

**Figure 4:**
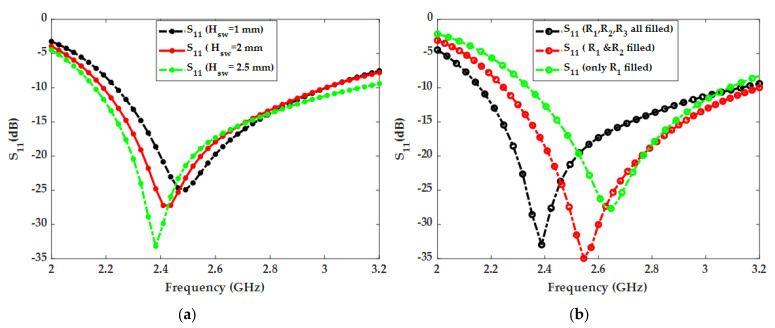
Effects of sea water on the S_11_ parameter. (**a**) Effects of sea water height on S_11_. (**b**) Effects of seawater radius on S_11_.

Figure 3 shows the impact of distilled water loading on the proposed water patch antenna performance. The impedance bandwidth was significantly improved by adding distilled water to the top resin container. Without distilled water loading, the antenna has a narrow bandwidth, even if the entire segmented radiator is filled with sea water. This parametric analysis shows that the loading section is one of the most important parts of the proposed antenna. The radiation efficiency also reached its peak value of 95% at 2.4 GHz with loading as shown in Figure 3b. In this section, the effects of sea water and the relevant parametric analysis are presented after the detailed analysis of distilled water loading. Sea water was used for the radiator part of the proposed antenna because of its conductivity. The height/thickness and radius of the seawater radiator in the proposed antenna were also varied to optimize the impedance bandwidth and frequency reconfigurability. Figure 4a shows the effect of seawater height on S_11_. As shown, the impedance bandwidth improves as the height is increased from 1 mm to 2.5 mm. Therefore, the optimized height of 2.5 mm was selected for the final design. Figure 4b shows the effect of seawater radius on the resonant frequency. As shown, the resonant frequency shifts from 2.6 GHz to 2.4 GHz as the radius is increased using the three sections of the transparent resin container. This behavior can be utilized to implement frequency reconfigurable antenna.

### Measurements Results

**Figure 5:**
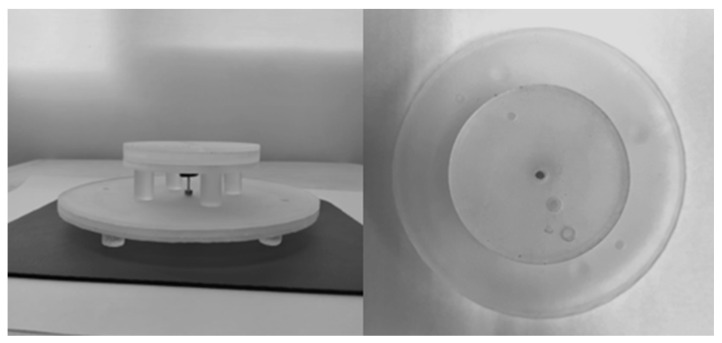
Fabricated prototype of the proposed antenna.

The proposed water antenna shown in Figure 5 was fabricated and measured to validate its design. For fabrication, transparent resin was used to fabricate each part of the antenna, such as a circular container used to hold distilled water, another container for the loading section, and a segmented radiator using a 3D printer. The 3D printer provides an easy and cheap way to make different containers made of different shapes. After printing, each part was washed for 15 min in an alcohol solution avoiding resin mixing with water. Some irregularities and roughness of the different shaped resin container can occur after printing which results in leakage of water because of fabrication tolerance. This can be effectively addressed through careful fabrication.

**Figure 6:**
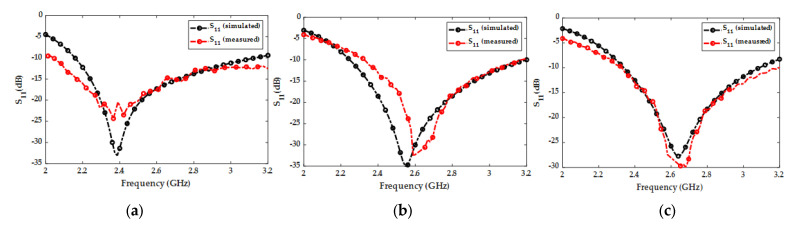
Simulated and measured S-parameters of the proposed antenna. (**a**) S_11_ responses when R_1_, R_2_, and R_3_ are filled. (**b**) S_11_ responses when R_1_ and R_2_ are filled. (**c**) S_11_ responses when only R_1_ is filled.

**Figure 7:**
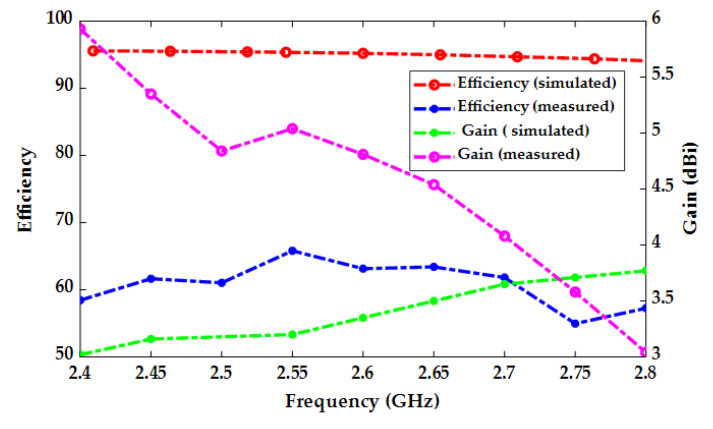
Simulated and measured efficiencies and gain of the proposed antenna.

The simulated and measured S_11_ parameters are plotted in Figure 6. As the antenna’s resonant frequency can be tuned by filling the segmented radiator with sea water from the top holes (through the loading part), each plot in Figure 6 represents a different sequence of sea water filling in each section (R_1_, R_2_, R_3_) of the segmented radiator. The results show a good agreement between the measured and simulated S_11_ parameter. The measured and simulated results for radiation efficiency and gain are illustrated in Figure 7. The measured maximum efficiency for the proposed antenna was 65% while the peak measured gain was 5.9 dBi.

**Figure 8:**
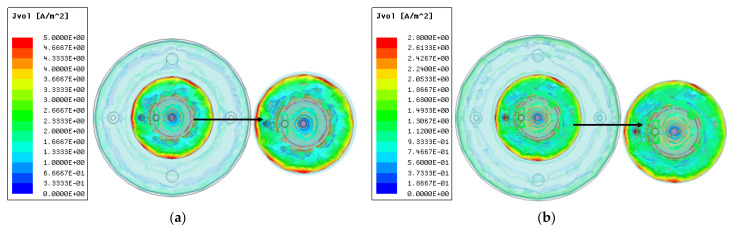
Current distribution of radiator at frequencies (**a**) 2.4 GHz, (**b**) 2.5 GHz.

Due to larger difference of the dielectric constants of the air and water, the electromagnetic energy propagates from the metal disk to the free space and then the edges of sea water radiator. A very week field exists inside the distilled water loading part, showing that the proposed antenna is not working in dielectric mode. Because the radiator has sea water with a conductivity, it can be observed that current is maximum at the edges of the radiator and hence this property has been utilized to reconfigure the frequency of the proposed antenna by filling/evacuating water from the segmented radiator. Figure 8 shows the current distribution of the radiator at two different frequencies.

**Figure 9:**
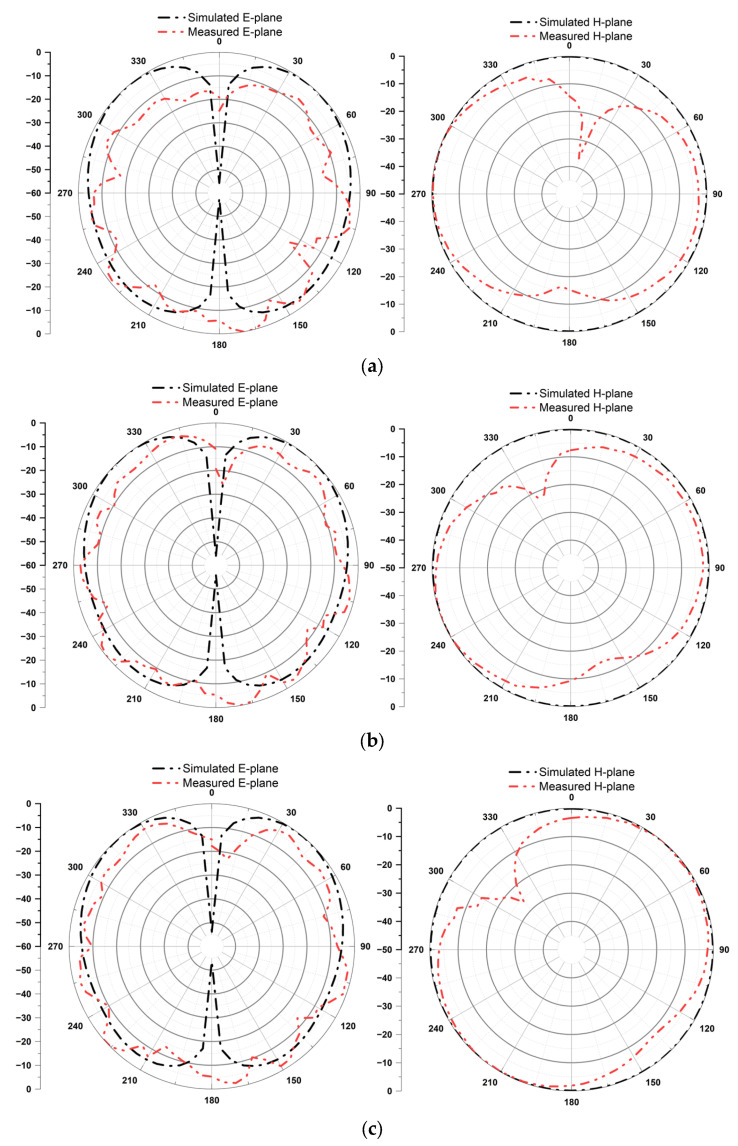
Simulated and measured E and H plane of the proposed antenna at (**a**) 2.4 GHz, (**b**) 2.5 GHz, and (**c**) 2.6 GHz.

Figure 9 shows the measured and simulated radiation patterns for E and H planes at 2.4, 2.5, and 2.6 GHz. The measured radiation pattern has been influenced by several factors, including temperature, air gaps within the antenna structure, variations in water conductivity, water leakage, and the use of adhesive to connect different antenna components. The performance of the proposed water antenna could be affected by significant changes in water properties like dielectric constant and loss tangent. At low frequencies the distilled water has high permittivity and low loss tangent, however at high frequencies the effect of permittivity decreases, and loss tangent is increased. The effect of environmental conditions such as temperature and the pressure on these water parameters is explained in [29,30]. Therefore, the temperature and the presence of air gaps in the antenna’s structure can also affect the antenna performance. It is worth mentioning that, in general, the electrical conductivity of water is increasing with an increase in temperature. However, it’s important to note that this change in conductivity and the resulting shift in frequency only happens when the temperature increases significantly, well beyond the standard laboratory temperature range of 25–35 °C. Additionally, the air gaps within the antenna structure are important for maintaining a constant water level inside the antenna. When these gaps are unintentionally sealed, some air, along with its pressure, can get trapped inside the antenna. This can lead to the water inside the antenna moving freely in various directions within differently shaped resin containers. This challenge becomes particularly significant when we’re trying to measure the 3D radiation pattern. In such cases, we observed higher radiation intensity in the direction where there is more water, and the measured radiation pattern is not ideal. This fluctuation in water volume affected various components of the antenna, including the ground, radiator, and loading section. The presence of air gaps and minor irregularities resulting from the fabrication process, which involved 3D printing with some surface roughness, contributed to these fluctuations. However, this issue can be effectively addressed through careful fabrication techniques.

**Figure 10:**
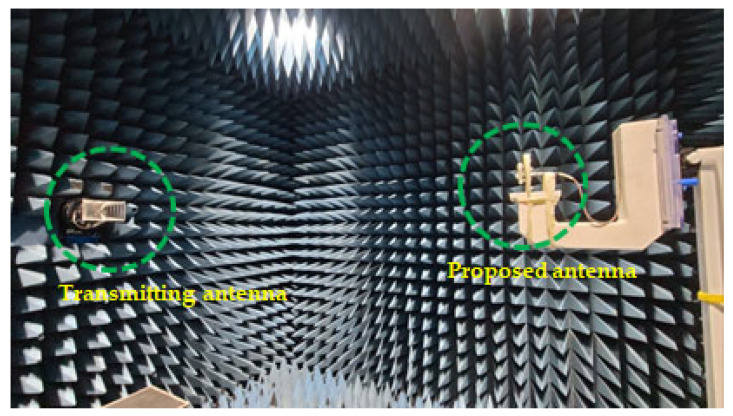
Fabricated prototype of the proposed antenna during far field measurements.

Radiation pattern investigations were conducted in a shielded millimeter-wave anechoic chamber room with a commercial ORBIT/FR far-field measurement system, as shown in Figure 10.

Performance comparison outcomes of some water-based antennas are listed in Table 2. In the table, the proposed water antenna responses are compared based on different parameters, such as size (in terms of wavelength) at the center frequency, bandwidth, efficiency, use of metallic ground (which restricts transparency), and the capacity of frequency tuning. The proposed antenna exhibits a medium size and features a transparent structure. In comparison to antennas cited in references [9,10,14], the distinctive feature of transparency is achieved through the utilization of plexiglass and water as materials. However, it is worth mentioning that glass, while offering superior transparency, is susceptible to unexpected cracks and breakage. The fabrication process of making different shaped containers from transparent resin using 3D printing is easy and cheap. It has low chances of cracks or breaking as compared to plexiglass. Similarly, the proposed antenna does not use metallic ground like a few listed antennas and hence leads to a fully transparent structure. Of particular significance, in contrasts to the other antennas discussed in the literature, the proposed antenna stands out for its exceptional efficiency and its remarkable ability to adjust its operating frequency through the deployment of a segmented radiator.

## 4. Conclusions

We presented herein a microfluidic and optically transparent water antenna, designed for flexible reconfiguration to operate at different frequencies. The antenna is fabricated through 3D printing, utilizing transparent resin and consists of three essential components: a ground formed by distilled water, a radiator incorporating sea water and a distilled water-loading segment. Notably, the radiator features distinct sections designated for sea water, which can be filled to effectively adjust the antenna’s resonant frequency within the range of 2.4 to 2.6 GHz. The use of distilled water loading significantly improved the antenna’s impedance, spanning from 2.10 to 3.15 GHz with a peak gain of 3.8 dBi and a maximum simulated radiation efficiency of 95%. This novel antenna configuration not only exhibits excellent performance but is also notable for its cost-effectiveness, moderate size, and complete optical transparency. Consequently, it presents a promising solution for potential applications involving transparent and flexible electronics in the future.

## Figures and Tables

**Table 1:** Optimized dimensions of the proposed antenna.

Parameter	Value (mm)	Parameter	Value (mm)
R_g_, H_g_	67, 6.5	R_r,_ R_w_	35, 33
R_gw_, H_gw_	65, 3	R_1_, R_2_, R_3_	33, 20, 7
t_1_, t_2,_ t_3_, t_4_	1.5, 2, 1, 2	H_l_, H_r_	6.5, 3
L_1_, L_2_, r	10, 15.5, 4	H_w_, H_sw_	3.5, 2.5

**Table 2:** Summary of comparison outcomes of our proposed with some other transparent antennas.

Ref.	Antenna Size	Material of Patch	Material of Ground	Impedance Bandwidth (%)	Efficiency (%)	Transparency	Container/ Substrate	Frequency Tuning
[5]	1.14λ_0_ × 1.14λ_0_ × 0.24λ_0_	Water	Copper	57.3%	52–84%	No	Acrylic tubes	No
[6]	1.06λ_0_ × 1.06λ_0_ × 0.06λ_0_	Water	Copper	8%	>70%	No	Plexiglass	No
[7]	1.95λ_0_ × 0.6λ_0_ × 0.25λ_0_	Water	Water	82.5%	43–60%	Yes	Transparent resin	No
[9]	1.94λ_0_ × 1.77λ_0_ × 0.47λ_0_	Water	Water	42.6%	67%	Yes	Plexiglass	No
[10]	1.35λ_0_ × 1.25λ_0_ × 0.35λ_0_	Water	Water	34.9%	75%	Yes	Plexiglass	No
[11]	0.04λ_0_ × 0.03λ_0_ × 0.01λ_0_	Water	Copper	68.9% (−6 dB)	12–75%	No	Acrylic plastic	Yes
[12]	2.0λ_0_ × 1.8λ_0_ × 0.16λ_0_	AgHT-8	AgHT-8	12%, 28.8%	>75%	Yes	Plexiglass	No
[14]	7.5λ_0_ × 2.4λ_0_ × 0.30λ_0_	Water	Water	35%	57–82%	Yes	Plexiglass	No
[Prop.]	6.73λ_0_ × 1.07λ_0_ × 0.25λ_0_	Water	Water	40%	80–95%	Yes	Transparent resin	Yes

λ_0_ refers to the wavelength in terms of the free space at the center operating frequency.

## Data Availability

The data presented in this study are available on request from the corresponding author.
